# Supplementary material for: Exploring commercial sex work among transgender women in Nepal: Contributors and stigma—A mixed-method study
Source: PLoS One. 2024 Dec 2;19(12):e0314619. doi: 10.1371/journal.pone.0314619 (PMC11611081; doi:10.1371/journal.pone.0314619)
Supplement: Attachment — Submitted filename: Response to Reviewers.docx [file pone.0314619.s004.docx]

Dear Editor,

We sincerely thank you and the reviewers for your valuable feedback and time dedicated to improving our manuscript. We have carefully considered each comment and have made revisions to address these suggestions. Below is a detailed response to each point raised:

Editor comment and author response

1) Please be consistent with the use of TGW and CSW throughout sometimes it is written as Transgender women and other times TGW, the same with commercial sex work

- We have ensured consistent use of terminology throughout the manuscript. Specifically, we have spelled out “Transgender women” and “commercial sex work/commercial sex workers” throughout the document to enhance clarity for readers and avoid any potential confusion. We have refrained from using the abbreviations TGW and CSW.

2) Please use the term commercial sex work rather than prostitution

- We have replaced the term "prostitution" with "commercial sex work" throughout the manuscript.

3) In the Introduction take out reference to trans-sexual - use transgender only

- In the introduction, we have removed the term "trans-sexual" and used "transgender" exclusively, as suggested.

4) In the introduction you write 'in comparison to female sex workers' maybe consider using the term 'cis-gendered women' rather than female.

- Thank you, We have replaced “female sex workers” with “cis-gendered women” to align with more accurate and inclusive terminology.

**Reviewers comment and author response**

Although the text presents the steps of the analytical process of qualitative data, the authors did not clarify which analysis technique was used or include a reference.

- We appreciate the time and effort that has been dedicated to strengthening our work. Thank you for notifying the missing information, we have now clarified that thematic analysis was used, along with an appropriate citation detailing the specific steps followed.

References published more than 5 years ago predominate.

- We acknowledge the feedback regarding the use of references predominantly older than five years. Due to the specificity of our research topic, recent studies are limited, and many current publications refer back to foundational sources. To ensure accuracy, we chose to cite original sources where relevant rather than secondary citations in more recent publications. We hope this approach is understandable given the context of our study. Thank you once again for the opportunity to improve our manuscript.
